# 100 years Journal of Comparative Physiology

**DOI:** 10.1007/s00360-024-01563-3

**Published:** 2024-06-22

**Authors:** Gerhard Heldmaier

**Affiliations:** Animal Physiology, Department of Biology, Philipps Universitaet, Marburg, Germany

The Journal of Comparative Physiology celebrates its 100th anniversary this year. It was founded in 1924 as “Zeitschrift für vergleichende Physiologie“ by Karl von Frisch and Alfred Kühn. In 1977 the journal language was changed to English and the title to “Journal of Comparative Physiology”. A few years later in 1984 the journal was split in part A and B. Since then Journal of Comparative Physiology A focusses on the publication of articles in neurophysiology and sensory physiology. Journal of Comparative Physiology B focusses on the publication of articles on metabolic physiology and environmental adaptation. Therefore, this year we also celebrate the 40th anniversary of Journal of Comparative Physiology A and Journal of Comparative Physiology B.

A review of the early history and significance of this journal was outlined on the occasion of the 90th anniversary of Journal of Comparative Physiology (Heldmaier et al. 2014). The present editorial focusses on recent developments and future perspectives of comparative physiology.

Research in comparative physiology is driven solely by human curiosity to understand animal life on earth. Animals live in water and on dry earth. Animals can creep, swim, run and fly. They inhabit shallow waters as well as the deep sea, dense forests, hot deserts, arctic ice, high mountains and areas with permafrost. Carey (2015) defined comparative physiology as “the exploration of physiological principles through examination of the functional diversity among animal species”. This diversity has evolved during the past 1 billion of years on the basis of common biochemical pathways and cellular structures. Selection pressure extended this common machinery to invent new ways of living and to adapt to the most different regions of the earth.

The unconceivable variety of animal life on earth can only be analysed by exemplary research on selected animal species. A comparison of animals adapted to different environmental conditions and from different phyla allows to discover general principles of the plasticity of physiological mechanisms, novel physiological mechanisms, as well as insights into principles of genetic adaptation. This comparative approach is reinforced by the discovery and analysis of extremes which, at first glance, appear to contradict existing knowledge or physical limits, like birds flying at altitudes above 9 000 m or life at subfreezing body temperatures in frozen frogs or hibernating Arctic squirrels (Scott et al. 2015; Costanzo 2019; Barnes 1989).

Studies on unknown physiological mechanisms are a major challenge because new methods are required to analyse the causality of physiological mechanisms. Fortunately, sensor technology, telemetric methods and computers rapidly improved during the past three decades which now allow experimental studies on unrestrained freely moving animals even by using satellite technology. At the same time cellular biochemistry and molecular genetics rapidly progressed and now allows insights into details of the physiological machinery of animals which were unthinkable 100 years ago. The past progress in our understanding of animal life through basic research underlines the significance of human ingenuity and freedom of research for the sustained progress in life sciences.

The genetic background of proteins and their organismic relevance is most conveniently disclosed by the study of laboratory animals because they can be genetically modified and can be maintained and studied in larger numbers. Knowledge gained from these studies can be approved by targeted approaches in other species or wild animals. Quite often this comparison sheds new light on physiological processes and environmental adaptation. Therefore comparative physiology needs tight cooperation with biochemistry, molecular genetics, evolutionary biology and ecology to understand the evolution of physiological mechanisms and to discover general principles in animal physiology.

Progress in comparative physiology also contributes to a better understanding of human biology and medicine. Diagnostics and medical treatments have substantially improved during the past 100 years, a development which is flagged by the Nobel Prizes for physiology and medicine. Research of many prize winners is based on basic research in animals, sometimes with an explicit comparative approach. Krogh (1929; Nobel Prize 1920), one of the pioneers of comparative physiology went even one step further, beyond the mere exploration of animal and human physiology, and recommended to use “animal models to answer questions that could not be answered in humans”, which is known as Krogh’s principle. This illustrates the dilemma of physiological research which always needs animal experiments to disclose the secrets of animal life and to use this knowledge for improvements of human life and medical improvements.

Since 1984 Journal of Comparative Physiology B published 729 articles on metabolic physiology of animals, ranging from Cnidaria through Chordata. Most heavily cited articles out of this period are reviews on glucose metabolism of fish (Polakof et al. 2012), on the specific dynamic action of food (Secor 2009), the toxicity of Dioxin (Mandal 2005) and suppression of ageing in naked mole-rats (Buffenstein 2008). This span of topics illustrates the broad coverage of Journal of Comparative Physiology B and its focus on organismic aspects in comparative physiology.

Citation analysis of a more recent period, the last 10 years (2013–2023), placed an article on the evolution of brown adipose tissue and nonshivering thermogenesis at the top of the interest scale (Oelkrug et al. 2015). This mechanism of heat generation was previously considered as a unique achievement of mammalian endothermy, caused by the action of UCP1 a unique protein in mammalian mitochondria. Genomic analysis showed that the gene for UCP1 is already present in fish, which revealed the evolution of endothermy in a completely different light. Further highlight articles during the recent period were reviews on thermal trait variation in lizards (Clusella-Trullas and Chown 2014) and the role of membrane lipids and polyunsaturated fatty acids for longevity in animals (Hulbert et al. 2014). The lizard article points to the impact of environmental change on animal diversity. The articles on brown adipose tissue and on animal longevity show that animal life is only fully understood if the organismic relevance of molecular properties is analysed and if these properties are placed into an evolutionary context.

The editorial board of Journal of Comparative Physiology B has changed during recent years. Ian Hume (Sydney, Australia) who started as an editor in 1996 resigned in 2018. Hannah Carey (Madison, WI, USA) started in 2005 and resigned at the end of 2023. She had a strong impact on the biochemical profile of the journal and also acted as Editor in Chief in 2020. Fritz Geiser (Armidale, Australia), Noga Kronfeld-Schor (Tel Aviv, Israel) served as editors in the years 2018 through 2019. Current editors of Journal of Comparative Physiology B are Kathrin Dausmann (ecophysiology; Hamburg, Germany), Bernd Pelster (aquatic respiration; Innsbruck, Austria), Phil Withers (comparative metabolism; Perth, Australia) since 2020. Most recently the editorial board was strengthened by Graham Scott (environmental adaptation; Hamilton, Canada), Todd Gillis (cardiovascular physiology; Guelph, Canada), and Martin Klingenspor (central control of metabolism; Munich, Germany).

The understanding of animal life requires knowledge about their genetic, biochemical, cellular and systemic properties. One might assume a hierarchy between these levels of complexity in the sense that genes contain the building and working plan for animal life, which is translated into proteins, metabolic pathways, cellular and morphological structures and physiological properties of animal life. This one-way organisation of life is certainly not adequate because comparative physiology revealed numerous interactions between these different levels of complexity. Environmental conditions affecting animal life and behaviour, induce compensatory regulation or adaptation on a short-term scale, as well as genetic adaptation and evolution on a long term scale. It is the unique strength of comparative physiology to integrate all these different actions to understand the hypercomplex causalities of animal life.

There are still large gaps in our understanding of animal physiological processes. These include in particular control functions of the nervous system, the endocrines, metabolic regulation, and the immune system. Cellular interactions on tissue level and the neural or endocrine control of these metabolic processes on the organismic level are only poorly understood. This underlines the continued need for research in comparative physiology in the future. One of the major gaps in animal physiology is our knowledge about sleep. All animals sleep, as far as we know, and spend a large portion of their lifetime in sleep. Humans sleep for about one third of all their lifetime. Sleep is essential because withdrawal of sleep has fatal consequences in animals and humans and may be lethal within a few days (Cirelli and Tononi 2008). Sleep is necessary but there is no clear answer to the question why animals sleep.

This gap of knowledge stimulated us to publish a special issue on the “Comparative Physiology of Sleep and Circadian Rhythms” on the occasion of the 100th anniversary of Journal of Comparative Physiology B. Vladyslav Vyazovskiy and Stuart Peirson gratefully edited this special issue which provides an overview about the occurrence of sleep and the into the comparative physiology of sleep. Russell Foster (University of Oxford), one of the pioneers of research in circadian rhythms and sleep introduced the topic and early development of this research area, and emphasized the necessity for basic research to further our understanding about sleep and its link with endogenous rhythms. Journal of Comparative Physiology B will continue to support basic research in animal physiology.
